# Dl‐3‐n‐Butylphthalide Promotes Cortical Angiogenesis via Akt/GSK‐3β Signaling in Ischemic Stroke Mice

**DOI:** 10.1002/cns.70698

**Published:** 2025-12-10

**Authors:** Lan Zhang, Shanshan Wei, Jian Zhang, Rong Chen, Jiangyong Miao, Lina Wang, Peipei Zhang, Wenyan Shang, Renhao Xu, Xiangjian Zhang, Cong Zhang

**Affiliations:** ^1^ Department of Neurology Second Hospital of Hebei Medical University Shijiazhuang Hebei People's Republic of China; ^2^ Hebei Key Laboratory of Vascular Homeostasis and Hebei Collaborative Innovation Center for Cardio‐Cerebrovascular Disease Shijiazhuang Hebei People's Republic of China; ^3^ The Key Laboratory of Clinical Neurology, Ministry of Education Hebei Medical University Shijiazhuang Hebei People's Republic of China; ^4^ Department of Neuromuscular Disease The Third Affiliated Hospital of Hebei Medical University Shijiazhuang Hebei People's Republic of China; ^5^ Department of Geriatrics Second Hospital of Hebei Medical University Shijiazhuang Hebei People's Republic of China

**Keywords:** Akt/GSK‐3β pathway, angiogenesis, dl‐3‐n‐butylphthalide, ischemic stroke, vasodilation

## Abstract

**Aims:**

Dl‐3‐n‐butylphthalide (NBP) is a novel agent for acute ischemic stroke. This study aimed to investigate its effects on cortical angiogenesis and vasodilation during stroke recovery.

**Methods:**

Mice underwent distal middle cerebral artery occlusion (dMCAO) and subsequently received NBP treatment. Therapeutic efficacy was measured by neurological deficits and infarct size. Angiogenesis was assessed by immunofluorescent staining. Laser speckle and two‐photon microscopy imaging were employed to evaluate dynamic changes in cortical cerebral blood flow and vascular structure in vivo. The modulation of the Akt/GSK‐3β signaling pathway was detected by western blotting.

**Results:**

NBP administration promoted neurological recovery and reduced infarct size in the subacute phase. It facilitated cerebral blood flow and vasodilation, enhanced angiogenesis as evidenced by increased BrdU^+^/CD31^+^ cells and improved astrocyte/pericyte coverage around microvessels. Moreover, the pro‐angiogenesis effect of NBP depends on the activation of the Akt/GSK‐3β pathway, and this effect is blocked by LY294002.

**Conclusion:**

In conclusion, NBP enhances recovery after ischemic stroke by promoting cortical angiogenesis and vasodilation through activation of the Akt/GSK‐3β pathway. These findings highlight its therapeutic potential for delayed intervention in ischemic stroke.

## Graphical abstract

NBP promotes post‐stroke angiogenesis by upregulating VEGF through activation of the Akt/GSK3β pathway. NBP enhances vascular maturation via increased pericyte and astrocyte coverage of cerebral microvessels. NBP facilitates cerebral blood flow and augments cortical vasodilation in ischemic stroke mice.

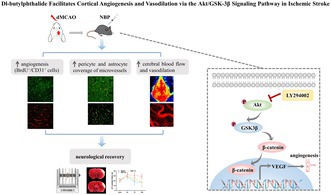

## 1 Introduction

Ischemic stroke remains the predominant cause of mortality and long‐term disability globally. Intravenous thrombolysis and endovascular thrombectomy represent effective interventions for acute ischemic stroke. However, constrained by the narrow therapeutic window or limited healthcare accessibility, the majority of patients fail to access timely reperfusion therapy, often leading to persistent neurological impairments. This reality underscores the pressing demand for novel neurorestorative strategies.

Among neurorestorative strategies, promoting angiogenesis has emerged as a particularly promising approach. Angiogenesis, defined as the formation of new blood vessels through branching from pre‐existing vasculature, represents a critical endogenous brain self‐repair mechanism following stroke [1]. Previous studies have demonstrated that angiogenesis initiates as early as 3–4 days post‐ischemia in the human brain, while in rodent models, this process typically becomes evident from 7 to 14 days following ischemic insult [1, 2, 3]. This process facilitates the restoration of blood flow and delivers essential nutrients and oxygen to ischemic brain tissue, ultimately promoting the recovery of damaged neurons and tissues [4]. Therefore, therapeutic augmentation of ischemia‐driven angiogenic processes could offer a viable pathway to improve functional outcomes after stroke.

Dl‐3‐n‐butylphthalide (NBP), a synthetic compound originally derived from celery seeds, is a novel therapeutic developed for ischemic stroke [5]. With its excellent blood–brain barrier (BBB) penetration, NBP exhibits a range of pharmacological effects, including mitochondrial protection [6], anti‐inflammatory activity [7], and promotion of neurogenesis [8]. Notably, previous studies have demonstrated that NBP significantly boosts angiogenic processes, enhances cerebral microcirculation, and improves functional recovery following ischemic injury [9, 10]. Nevertheless, the precise molecular mechanisms underlying NBP's pro‐angiogenic effects remain incompletely understood. In a previous study, Song H. et al. demonstrated that Zhuli Decoction combined with NBP provides neuroprotection against CO‐induced brain injury by activating the PI3K/Akt/GSK‐3β signaling pathway [11]. Given its established neuroprotective roles, we hypothesized that NBP may also facilitate post‐stroke angiogenesis through the Akt/GSK‐3β pathway, a key regulator of vascular remodeling. Therefore, this study aimed to investigate whether NBP enhances angiogenesis and vasodilation following ischemic stroke and to elucidate the potential involvement of the Akt/GSK‐3β pathway. Furthermore, we employed two‐photon microscopy to, for the first time, dynamically visualize the structural changes in cortical vasculature in live mice following NBP treatment.

## 2 Methods

### 2.1 Animals

Adult male C57BL/6 mice (20–25 g; 8–12 weeks) were obtained from Vital River Laboratory Animal Technology Co. Ltd. (Beijing, China). The mice were maintained under a 12/12 h light/dark cycle and ideal temperature (22°C ± 3°C) and humidity (60% ± 5%) with plenty of food and water. All procedures were approved by the Animal Care and Use Committee of the Second Hospital of Hebei Medical University (Permit No. HMUSHC‐130318) and the National Institutes of Health Guide for the Care and Use of Laboratory Animals (NIH Publication No. 80–23, revised in 1996).

### 2.2 Establishment of Ischemic Stroke Model

To establish the ischemic stroke model, mice underwent permanent distal middle cerebral artery occlusion (dMCAO) surgery [12]. Following intraperitoneal anesthesia with avertin (0.4 g/kg; Sigma), the surgical procedure began with permanent ligation of the right common carotid artery (CCA). Subsequently, a 1‐cm vertical skin incision was made between the right eye and ear to expose the temporal muscle and skull, and then the temporal muscle was divided to reveal the skull. After identifying the right middle cerebral artery (MCA) through the skull, a craniotomy was created over its bifurcation. Finally, the artery was coagulated between its cortical branches and the lateral striate arteries to achieve complete occlusion. In sham‐operated mice, the CCA and distal MCA were surgically exposed, but without ligation or coagulation.

The perioperative mortality rate was less than 5%, with primary causes including accidental subarachnoid hemorrhage and incorrect anesthesia. To ensure the stability of the model, we adopt the following exclusion criteria: any subarachnoid hemorrhage during surgery; operation time exceeding 15 min; recanalization of the MCA after two electrocoagulation attempts resulting in only transient occlusion.

### 2.3 Drug Administration

Dl‐3‐n‐butylphthalide (NBP; purity > 99.5%, Shijiazhuang Pharmaceutical Co. Ltd., China) was dissolved in a 0.5% Tween‐80 solution. Mice received daily intraperitoneal injections of NBP at doses of 10 or 20 mg/kg, beginning on the first postoperative day until sacrifice or continuing for 13 consecutive days.

LY294002 (MCE, Shanghai, China) was dissolved in a 1% DMSO solution and administered daily by intraperitoneal injection at a dose of 40 mg/kg 1 h before NBP. The optimal dose of LY294002 was established based on the relevant literature [13, 14].

Bromodeoxyuridine (BrdU; Sigma‐Aldrich), dissolved in 0.9% saline at a dose of 50 mg/kg, was administered intraperitoneally once daily beginning on day 1 after dMCAO until sacrifice or for 13 consecutive days.

### 2.4 Experimental Design and Animal Grouping

Mice were randomly allocated to their respective groups using a computer‐generated random number sequence. In the initial experiment, mice were randomly allocated to five groups: Sham, Sham + NBP (Sham +20 mg/kg NBP), MCAO, NBP‐L (dMCAO +10 mg/kg NBP), and NBP‐H (dMCAO +20 mg/kg NBP). Sham and MCAO groups were administered an equal volume of 0.5% Tween‐80 solution. These groups were used to evaluate neurological deficits and determine the optimal dose of NBP. For subsequent research on angiogenesis, mice were reassigned to four groups: Sham, Sham + NBP, MCAO, and NBP (dMCAO +20 mg/kg NBP).

For subsequent mechanistic studies, mice were randomly assigned to several groups: Sham (Sham +1% DMSO +0.5% Tween‐80), MCAO (dMCAO +1% DMSO +0.5% Tween‐80), NBP (dMCAO +20 mg/kg NBP + 1% DMSO), and NBP + LY (dMCAO +20 mg/kg NBP + 40 mg/kg LY294002).

The sample size (*n* = 4–6 per group) was chosen based on our previous experience and similar studies published in the field [15, 16], which demonstrated that this number of animals was sufficient to achieve statistically significant results for the primary endpoints. To ensure objective and unbiased assessment, all outcome evaluations were performed by investigators who were blinded to the group allocations. The personnel responsible for drug administration were distinct from those conducting the outcome assessments.

### 2.5 Neurobehavioral Tests

Neurological function was evaluated with the Rota‐rod, Adhesive Removal, Modified Neurological Severity Score (mNSS), and CatWalk tests prior to dMCAO on days 3, 7, and 14 post‐surgery.

Sensorimotor function was evaluated using the Rota‐rod test. Following baseline training, mice were placed on an accelerating rod (4 to 40 rpm over 4 min). The latency to fall was recorded over three trials, and the mean latency was used for statistical analysis.

The Adhesive Removal test was performed to evaluate sensorimotor deficits and postural asymmetry following ischemia. Firstly, two adhesive patches (0.3 × 0.4 cm^2^) were placed bilaterally on the forepaws with consistent pressure, and the order of tape placement (left or right) was randomized across animals and testing sessions. Following placement, mice were transferred to a Perspex box. Finally, the latency for the mouse to first contact the tape and then completely remove it from each paw was recorded separately within a 2‐min trial.

The mNSS evaluates motor, sensory, reflex, and balance functions, scored on a 0–18 scale (normal function, 0; maximal neurological deficit, 18). Higher scores reflect greater neurological impairment.

Walking performance was evaluated using the CatWalkXT system (v10.6, Noldus) as previously reported. Mice walked freely on an illuminated glass walkway, with three successful trials per mouse. The criterion for a passable run was characterized by steady locomotion along the corridor, absent of any cessation of movement or episodes of fur‐licking. Footprints were recorded with a camera, and the subsequent analysis was conducted using the CatWalkXT software. The following parameters of the left front limb (the limb affected by cerebral ischemia) were evaluated: run speed (mm/s) and stride length (mm).

### 2.6 Infarct Volume and Brain Atrophy

Infarct size was measured via TTC staining on day 7 post‐surgery. Brains were sectioned coronally into seven 1‐mm slices and stained with 2% TTC for 20 min at 37°C prior to fixation in 4% PFA. Infarct volumes were quantified using ImageJ. Infarct volumes were calculated after correcting for edema, using the formula: %HLV = {[total infarct volume—(the volume of intact ipsilateral hemisphere—the volume of intact contralateral hemisphere)] / contralateral hemisphere volume} × 100%.

Brain atrophy was assessed using the cortical width index (CWI) on day 14 after stroke [17]. Following transcardial perfusion with 4% PFA, brains were harvested and imaged using a digital camera (AxioCam, Zeiss). Cortical widths from midline to lateral edge were measured bilaterally using ImageJ. The CWI was defined as the ratio of ischemic ipsilateral/contralateral cortical width × 100%.

### 2.7 Measurements of Cortical Cerebral Blood Flow (CBF)

Cortical CBF dynamics were assessed in real time using laser speckle contrast imaging (LSCI; Periscan PIM 3 System, Perimed, Sweden). To capture the dynamic progression of post‐ischemic cerebral perfusion, including the acute phase, as well as key periods of repair, measurements were taken at baseline (pre‐surgery), immediately after surgery, and on post‐operative days 7 and 14. Animals received analgesic (buprenorphine, 0.05 mg/kg, subcutaneous) to minimize peri‐procedural pain. The skull was exposed via a midline parietal incision, and whole‐brain scans were acquired bilaterally using the PIM3 system at a fixed distance of 10 cm above the skull. For quantitative analysis, two identical regions of interest (ROIs) were drawn over the ischemic and corresponding contralateral cortical areas. The relative CBF was calculated as a percentage of the pre‐dMCAO baseline using PIMSoft.

### 2.8 Two‐Photon Microscopy Imaging (TPMI)

TPMI was employed to evaluate changes in microvascular diameter in the cortex. Mice were implanted with a cranial window (5 × 3 mm) and allowed to recover for 2–4 weeks before imaging. FITC‐dextran (SLCC2644, Sigma Aldrich, USA) was administered through the retro‐orbital sinus for vascular labeling, and images were acquired at baseline and on days 1, 3, 5, 7, and 14 using a ZEN 2.1 SP3 system. Vascular diameter was analyzed with ImageJ.

### 2.9 Gelatin‐Ink Perfusion

Vessel density was assessed in ink‐gelatin‐labeled brain sections. Mice were perfused with a mixture of 4% gelatin and 10% black ink following PFA fixation. After perfusion, mice were placed prone at 4°C overnight for gelatin solidification. Brains underwent postfixation in 4% PFA, soaking in 30% sucrose, and were finally cut into 100 μm thick coronal sections. For quantitative analysis, three non‐overlapping coronal sections were selected between bregma +1.42 mm and −0.82 mm. A standardized rectangular ROI was placed in the ischemic penumbra of the cortex. Microvascular density was calculated as the percentage area of ink‐labeled vessels using ImageJ.

### 2.10 Immunofluorescence Staining

Mice were perfused transcardially with ice‐cold PBS followed by 4% PFA. We collected brains and postfixed them overnight in 4% PFA, followed by 30% sucrose for 48 h, and then prepared 15 μm thick coronal sections using a cryotome (Thermo Fisher Scientific, USA). For BrdU immunostaining, sections underwent HCl denaturation (2 N, 37°C, 30 min), boric acid neutralization (0.1 M, pH 8.5, 10 min), blocking in PBS with 0.3% Triton X‐100 and 10% normal donkey serum (1 h, room temperature), and primary antibody incubation (overnight, 4°C). The primary antibodies used were listed in Table S1. The following day, sections were incubated with species‐appropriate fluorescent secondary antibodies (Alexa Fluor 405, 488, or 594 conjugated donkey anti‐sheep, rat, mouse, or rabbit IgG; 1:500, Jackson ImmunoResearch, Germany) for 2 h at 37°C. Negative controls were performed by omitting the primary antibody, and no specific staining was observed. Quantitative analysis was performed on coronal sections between bregma +1.42 mm and −0.82 mm, with all images processed and analyzed using ImageJ.

### 2.11 Western Blotting

Total protein was isolated from the ipsilateral cerebral cortex with a Total Protein Extraction Kit (Applygen Technologies Inc., Beijing, China), and concentrations were quantified via a BCA Assay Kit (Novagen, Madison, WI, USA). 50 μg protein samples were separated via SDS‐PAGE and electrotransferred to PVDF membranes (Millipore, USA). The membranes were blocked with blocking buffer for 1 h at room temperature and incubated with primary antibodies overnight at 4°C, followed by IRDye 800‐conjugated secondary antibodies (goat anti‐rabbit or anti‐mouse; 1:8000; Rockland, Gilbertsville, PA) for 1 h at room temperature. A complete list of all primary antibodies is provided in Table S2. The band intensities of target proteins were normalized to the loading control, β‐actin. Band intensities were measured using an Odyssey infrared scanner (LI‐COR Bioscience, USA), and spectral intensity analysis was performed using ImageJ.

### 2.12 Statistical Analysis

All quantitative data were performed with SPSS 26.0 (IBM, USA) and are expressed as mean ± SEM. For each dataset, normality was confirmed using the Shapiro–Wilk test, and homogeneity of variance was confirmed using Levene's test. As all datasets met these parametric assumptions, subsequent analyses were performed using parametric tests. Group comparisons were made using the unpaired Student's t‐test (two groups) or one‐way ANOVA with LSD post hoc test (multiple groups). For longitudinal data such as neurobehavioral outcomes, CBF measurements, and vascular diameter, repeated‐measures ANOVA was used to analyze the effects of time and treatment, as well as their interaction. When a significant interaction was found, a multiple comparisons test was used for post hoc analysis. *p* < 0.05 was considered significant.

## 3 Results

### 3.1 NBP Improved Neurological Function and Reduced Histological Damage After Stroke

**FIGURE 1:**
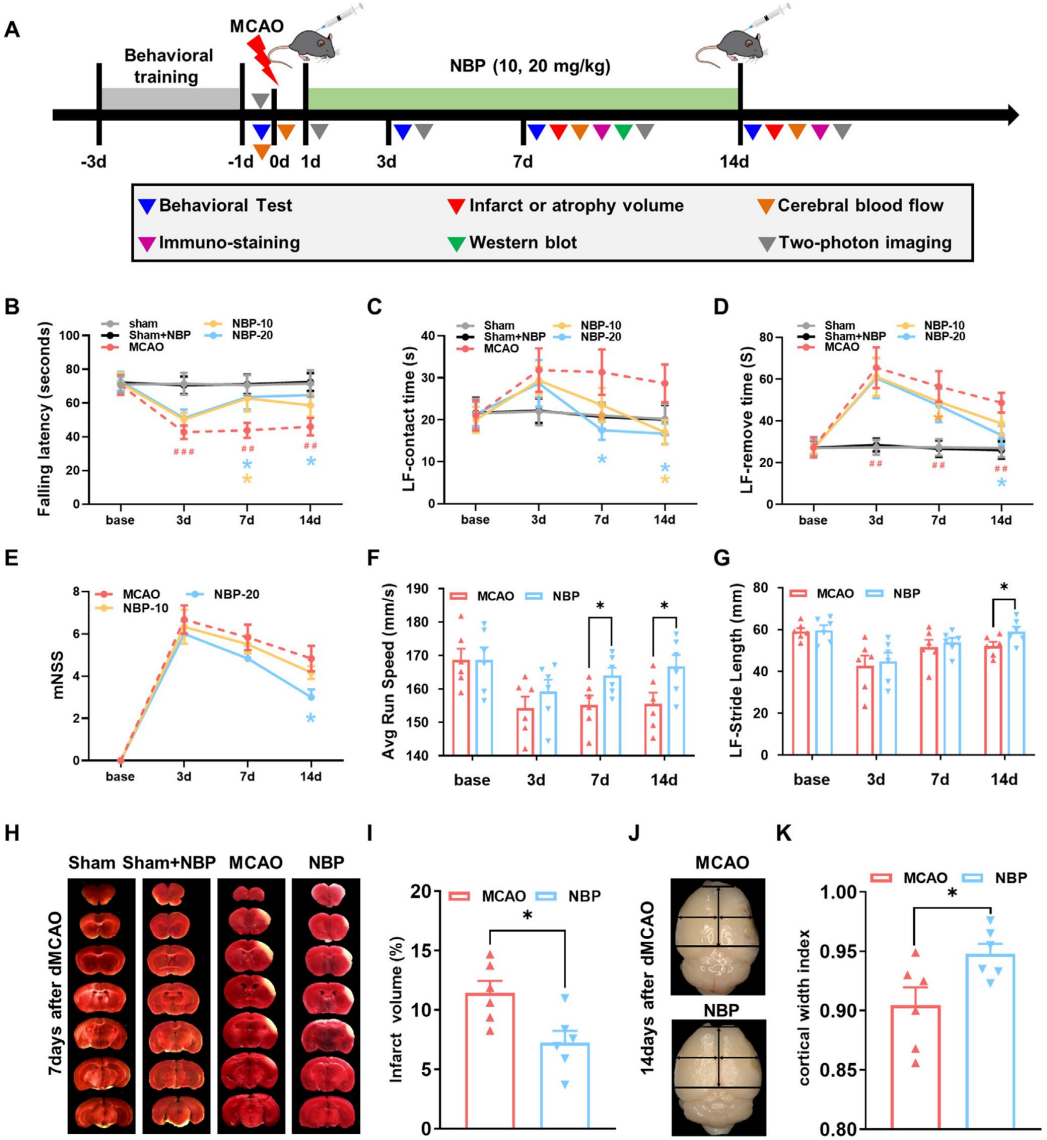
NBP facilitated functional neurological recovery and mitigated cerebral damage following stroke. (A) Experimental design schematic. (B‐G) Rota‐rod, adhesive removal, mNSS, and CatWalk test on days 0 (baseline), 3, 7, and 14 after stroke. (H, I) Representative TTC‐stained brain sections on day 7 and quantitative assessment of infarct volume. (J, K) Representative images on day 14 and quantitative analysis of cortical width index. *n* = 6, *p < 0.05 versus MCAO group, ##*p* < 0.01, ###*p* < 0.001 versus sham group.

The experimental design is illustrated in Figure 1A. To assess the neuroprotective properties of NBP, mice subjected to dMCAO were treated with 10 or 20 mg/kg NBP. Prior to ischemia, all mice exhibited normal neurological function. On day 3 after dMCAO, all groups displayed significant and comparable neurological deficits (Figure 1B–G). Rota‐rod testing showed significant neurological improvement in both NBP‐treated groups on day 7, but only the high‐dose group sustained improvement on day 14 compared to the MCAO group (Figure 1B). In the Adhesive Removal test, 20 mg/kg NBP significantly reduced both contact time on days 7 and 14 and removal time on day 14 after stroke. In contrast, the lower 10 mg/kg dose only improved contact time on day 14 and had no effect on removal time (Figure 1C,D). mNSS analysis revealed a significant reduction in neurological deficit scores on day 14 following 20 mg/kg NBP treatment, whereas the 10 mg/kg dose had no significant effect (Figure 1E). These findings collectively identify 20 mg/kg as the most effective dose for further study. NBP‐treated mice exhibited significant improvements in gait dynamics, as evidenced by increased LF‐run speed on days 7 and 14, as well as LF‐stride length on day 14 post‐stroke (Figure 1F,G). This indicates that NBP not only helped with basic tasks but also restored more complex walking patterns.

To assess histological damage, cerebral infarction volume and CWI were measured (Figure 1H–K). NBP lowered infarct volume on day 7 and raised CWI on day 14 post‐stroke compared to the MCAO group (Figure 1I,K). On a structural level, this means NBP treatment led to a smaller area of brain death and better preservation of brain tissue over time.

### 3.2 NBP Facilitates Focal Angiogenesis Following Stroke

**FIGURE 2:**
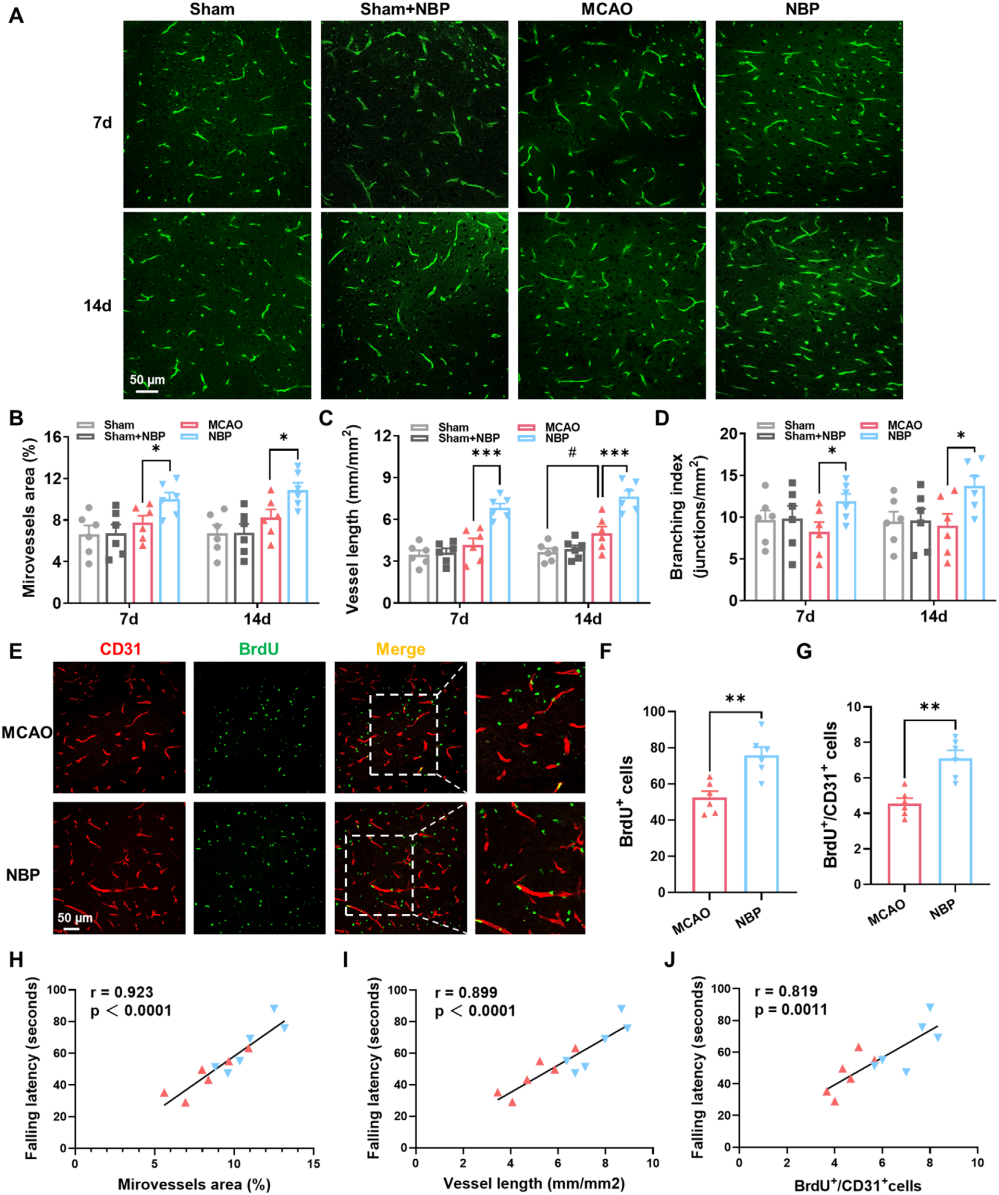
NBP augmented cerebral angiogenesis following dMCAO. (A) Representative CD31^+^ microvascular images on days 7 and 14. (B‐D) Quantitative analysis of microvessel density, total vessel length, and branching index. (E) Immunofluorescence double‐labeling of BrdU^+^ (green) and CD31^+^ (red) cells in the peri‐infarct cortex on day 14 post‐stroke. (F, G) Quantification of BrdU^+^ and BrdU^+^/CD31^+^ cells. (H‐J) Pearson correlation of microvascular parameters (microvessel area, vessel length, or BrdU^+^/CD31^+^ cells) and the residence time of Rotarod test on day 14 post‐MCAO. *n* = 6, *p < 0.05, **p < 0.01, ***p < 0.001 versus MCAO group; #*p* < 0.05 versus sham group.

To determine the pro‐angiogenic effects of NBP, we performed immunostaining for CD31^+^ endothelial cells (Figure 2A). Microvessel density, vessel length, and branching index were significantly elevated in NBP‐treated mice relative to MCAO‐treated mice (Figure 2B–D). Endothelial cell proliferation, assessed by BrdU^+^/CD31^+^ double labeling, was also significantly higher in the NBP group than in the MCAO group (Figure 2E–G). Crucially, these angiogenic parameters positively correlated with rotarod performance on day 14 (Figure 2H–J). This key finding suggests that the mice with more new vessel growth were also the ones with better motor coordination, providing a strong link between vascular repair and neurological recovery.

### 3.3 NBP Improves Astrocyte and Pericyte Coverage of Cortical Microvessels After Stroke

**FIGURE 3:**
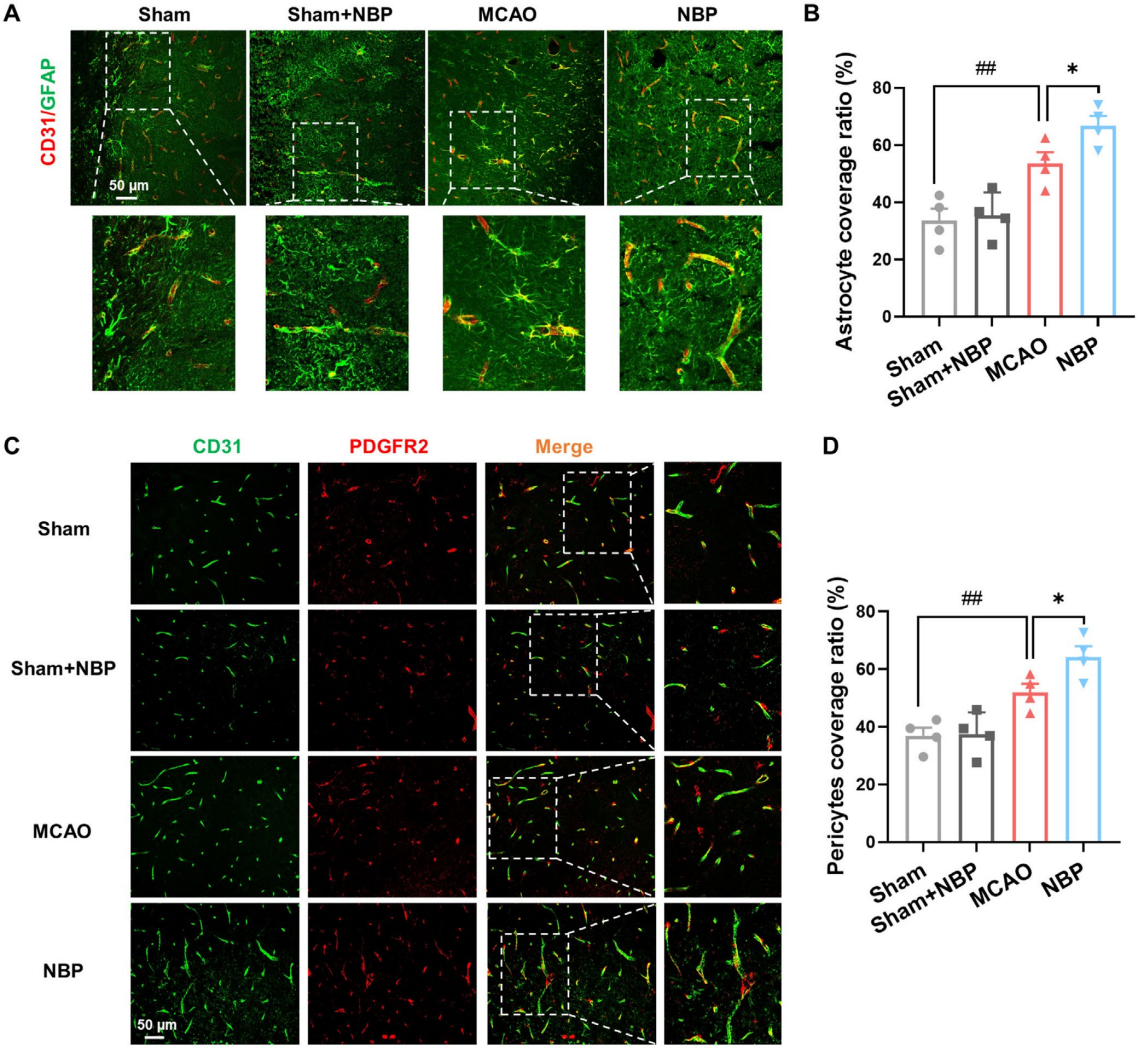
NBP improves astrocyte and pericyte coverage of cortical microvessels after stroke. (A) Representative immunofluorescence images of CD31 (red) and GFAP (green) on day 14 post‐stroke. (B) Quantitative analysis of astrocytic coverage around microvessels. (C) Representative immunofluorescence images of CD31 (green) and PDGFR2 (red) on day 14 post‐stroke. (D) Quantitative analysis of pericytes coverage around microvessels. *n* = 4, *p < 0.05 versus MCAO group; ##*p* < 0.01 versus sham group.

In stroke recovery, astrocytes and pericytes are essential for neurovascular remodeling. Compared to the sham group, the astrocyte and pericyte coverage ratio around microvessels was elevated in the MCAO group on day 14 after stroke, and NBP treatment further enhanced this coverage (Figure 3A–D). These findings strongly support the significant role of NBP in stimulating vascular remodeling after stroke.

### 3.4 NBP Improved Cerebral Reperfusion After Stroke

**FIGURE 4:**
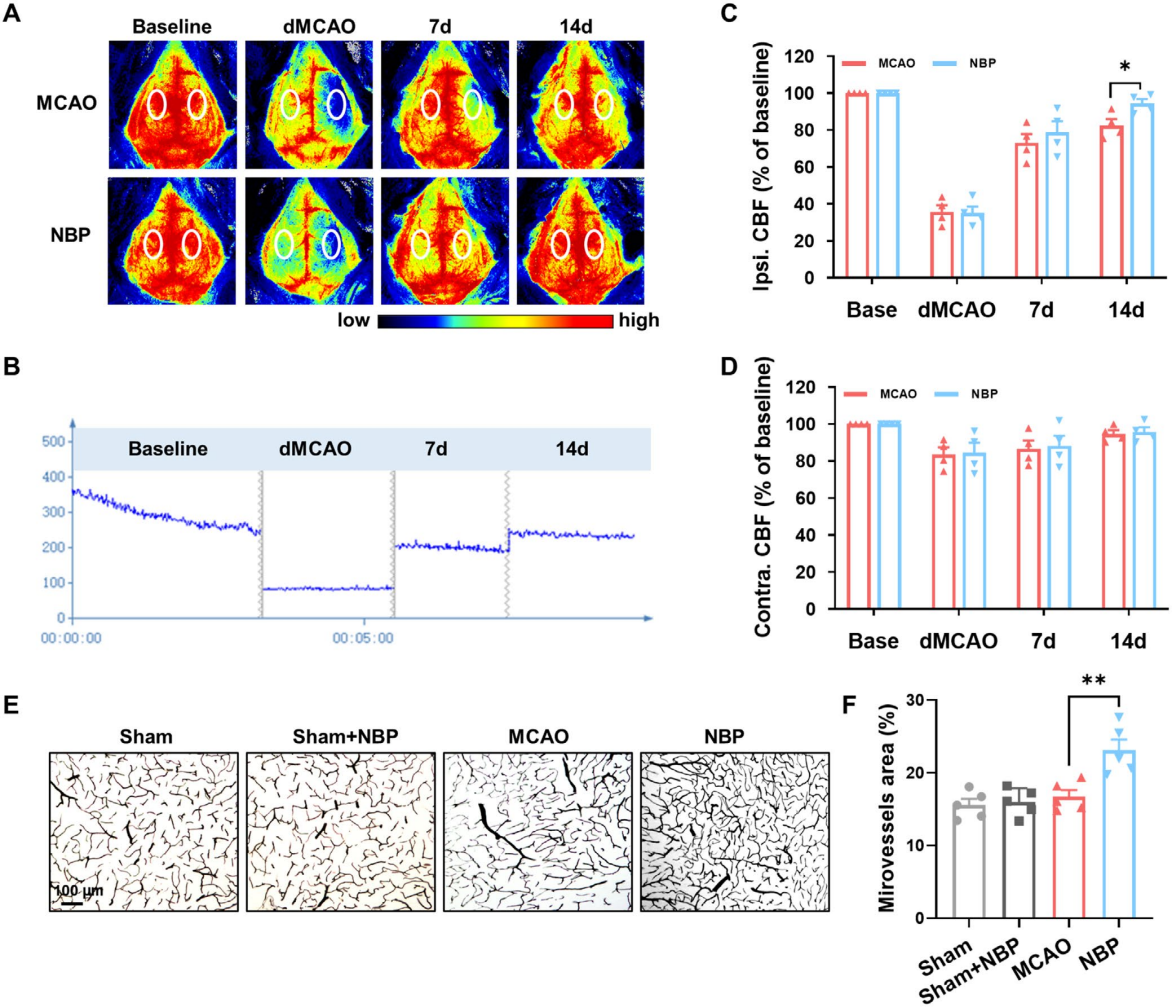
NBP treatment improved cortical CBF following ischemic stroke. (A) LSCI of the cerebral cortex from the MCAO and NBP groups. (B) A representative example of CBF fluctuations in the infarct region before and following dMCAO. (C, D) Quantitative CBF alterations (% of baseline) in the ipsilateral and contralateral cortex within the ROI (*n* = 4). (E) Gelatin‐ink angiograms of the ischemic penumbra area on day 14. (F) Quantitative analysis of microvessel density (*n* = 5). *p < 0.05, **p < 0.01 versus MCAO group.

Spatiotemporal CBF variations were monitored using LSCI to assess the impact of NBP on cerebral perfusion (Figure 4A). Immediately after dMCAO, CBF in the ipsilateral hemisphere was similarly reduced in both the MCAO and NBP groups to ~35% of baseline (Figure 4B,C). Although no significant differences in ipsilateral CBF were observed between the NBP and MCAO groups on day 7, the NBP group showed a significant improvement by day 14 post‐stroke (Figure 4C). Contralateral cerebral reperfusion remained unchanged across both groups (Figure 4D). Consistent with this functional improvement, a gelatin‐ink perfusion assay on day 14 revealed that NBP‐treated mice had a significantly higher microvessel density in the peri‐infarct zone compared to the MCAO group (Figure 4E,F). This provides direct visual evidence that the improved blood flow was due to a denser network of functional vessels in the recovering brain tissue.

### 3.5 NBP Upregulated VEGF Expression by Modulating the Akt/GSK‐3β/β‐Catenin Pathway

**FIGURE 5:**
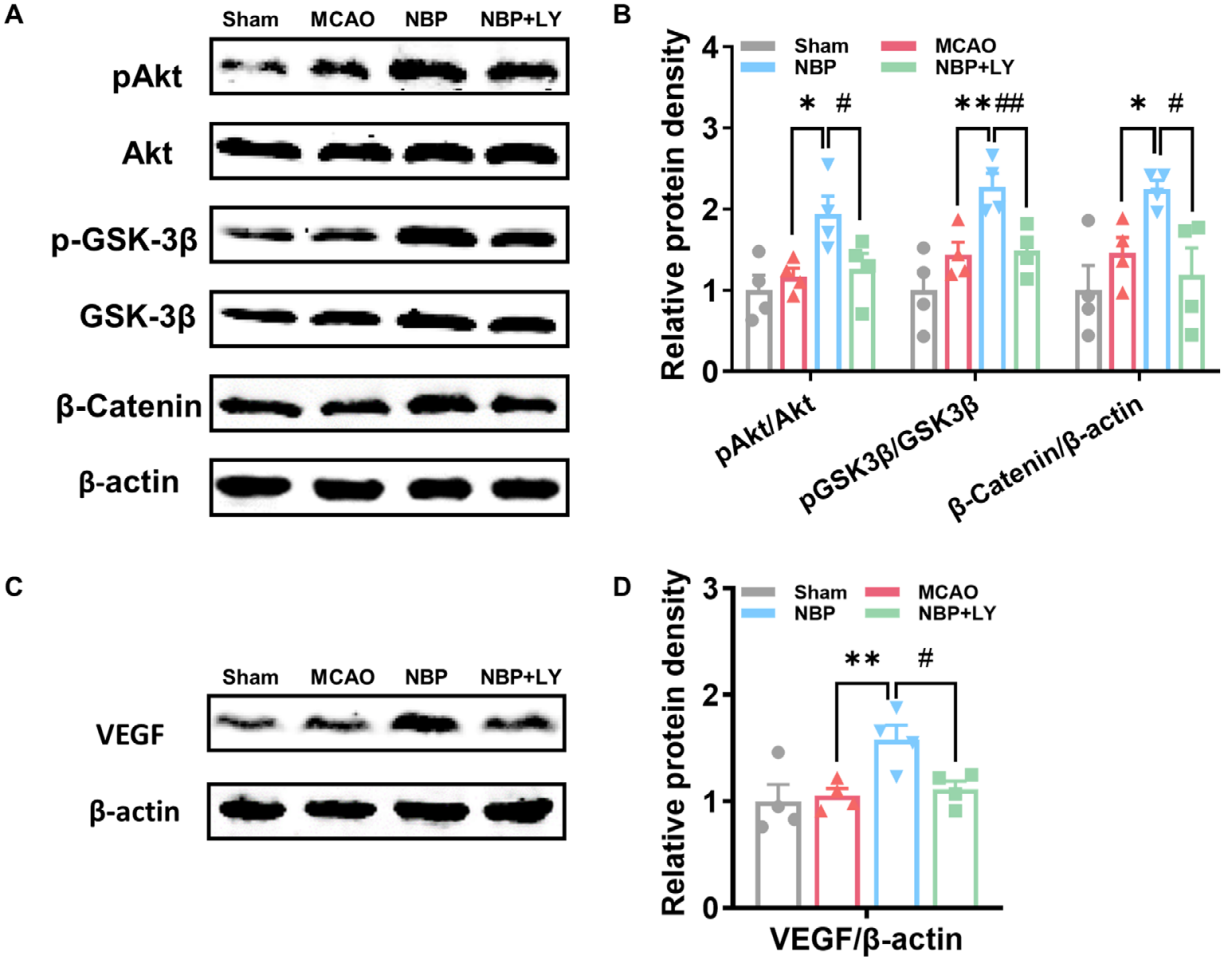
NBP activated the Akt/GSK‐3β signaling pathway, whereas this effect is blocked by co‐treatment with LY294002. (A) Representative images of p‐Akt, Akt, p‐GSK‐3β, GSK‐3β, and β‐catenin expression in the peri‐infarct region on day 7 post‐stroke. (B) Quantitative analysis of p‐Akt/Akt, p‐GSK‐3β/GSK‐3β, and β‐catenin. (C) Representative images of VEGF expression in the peri‐infarct region on day 7 post‐stroke. (D) Quantitative analysis of VEGF. *n* = 4, *p < 0.05, **p < 0.01 versus MCAO group, #*p* < 0.05, ##*p* < 0.01 versus NBP group.

To elucidate the mechanism of NBP‐induced angiogenesis, we found that NBP treatment enhanced Akt and GSK‐3β phosphorylation and reduced β‐catenin protein levels on day 7 (Figure 5A,B). This activation was accompanied by an upregulation of the pro‐angiogenic factor VEGF (Figure 5C,D), and both effects were effectively blocked by LY294002, a specific PI3K/Akt pathway inhibitor (Figure 5A–D). Together, these findings indicate that NBP promotes angiogenesis by activating the Akt/GSK‐3β signaling cascade, which in turn drives the production of VEGF.

### 3.6 NBP‐Mediated Cerebrovascular Repair and Neural Recovery Were Inhibited by LY294002

**FIGURE 6:**
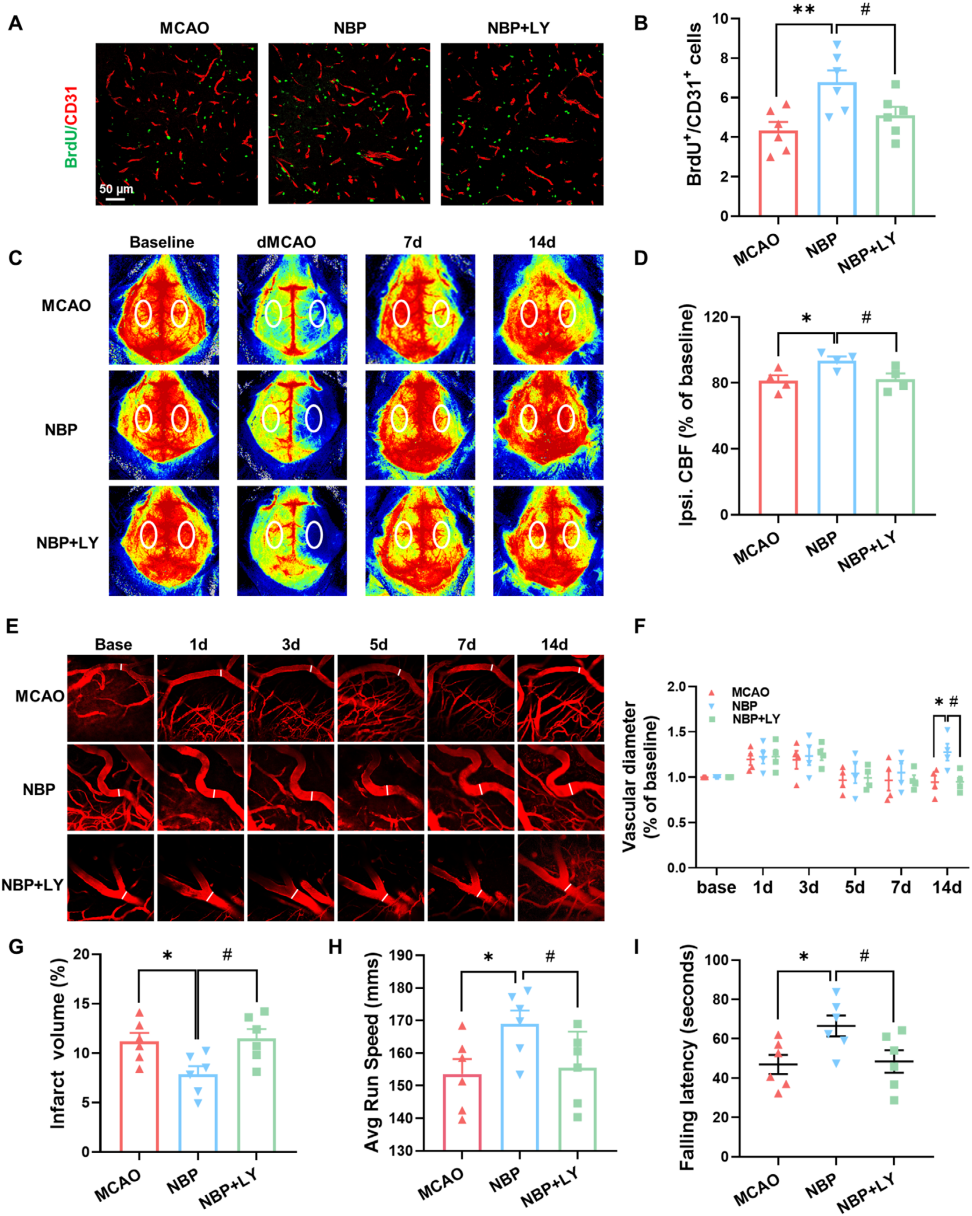
Akt/GSK‐3β pathway inhibition reversed NBP‐induced angiogenesis, vasodilation, and neural functional recovery. (A) Representative double‐labeling of BrdU^+^ (green) and CD31^+^ (red) cells on day 14. (B) Quantification of BrdU^+^/CD31^+^ cells (*n* = 6). (C) LSCI of the cerebral cortex from the MCAO, NBP, and NBP + LY groups. (D) Quantitative CBF alterations (% of baseline) in the ipsilateral cortex (*n* = 4). (E) In vivo two‐photon microscopy images of cortical vasculature projections in MCAO, NBP, and NBP + LY mice before and after stroke. (F) Quantification of vascular diameter (% of baseline) (*n* = 4). (G) Quantitative assessment of infarct volume on day 7 (*n* = 6). (H, I) Rota‐rod test and CatWalk test on day 14 after stroke (*n* = 6). *p < 0.05, **p < 0.01 versus MCAO group, #*p* < 0.05 versus NBP group.

To evaluate the vascular impact of LY294002, we administered LY294002 to sham‐operated mice (Sham + LY group) and assessed key vascular parameters. Our results showed that LY294002 administration did not cause significant vascular alterations in the healthy brain of sham animals (Figure S1A–H). To elucidate the role of the Akt/GSK‐3β pathway in NBP‐induced cerebrovascular repair and recovery, we administered LY294002 in the stroke model. The NBP‐mediated increases in BrdU^+^/CD31^+^ cells and CBF were markedly suppressed by LY294002 co‐treatment (Figure 6A–D). We utilized TPMI to examine the temporal dynamics of cortical microvascular diameter following NBP administration in living mice (Figure 6E). Vessel diameter showed no significant difference between groups until day 14 post‐stroke, when NBP resulted in a significantly greater diameter, an effect reversed by LY294002 (Figure 6F). Consistently, LY294002 also abolished NBP‐induced functional recovery on day 14 (Figure 6G–I). Collectively, these results demonstrate that NBP facilitates post‐stroke angiogenesis, vasodilation, and functional restoration through Akt/GSK‐3β pathway activation.

## 4 Discussion

Our findings reveal that NBP treatment enhances angiogenesis following ischemic stroke by upregulating VEGF via the Akt/GSK‐3β signaling pathway. While prior studies have established NBP's pro‐angiogenic role, our work provides several critical advancements. We uniquely demonstrate that NBP promotes vascular maturation through increased pericyte and astrocyte coverage of cerebral microvessels, a key step for functional stability. Furthermore, using advanced in vivo imaging (TPMI), we provide the first direct evidence that it drives significant vasodilation, linking structural remodeling to improved hemodynamics. It is important to note that these beneficial effects are dose‐dependent, as only the 20 mg/kg dose, not the 10 mg/kg, yielded a significant and sustained recovery. Therefore, we focused our mechanistic studies on this optimal dose, which revealed that its superior efficacy is linked to robust activation of the Akt/GSK‐3β pathway and enhanced vessel maturation.

Following ischemic insult, the ischemic penumbra rapidly secretes angiogenic factors that initiate and modulate the generation of new blood vessels [18]. This vascular remodeling process reestablishes cerebral perfusion to ischemic penumbral regions, rescuing metabolically active but underperfused neurons from delayed death. Newly formed vessels deliver oxygen, glucose, and trophic factors [19], creating a microenvironment conducive to neural repair. Post‐stroke angiogenesis modulates axonal regeneration and neurogenesis by promoting neural stem/progenitor cells' proliferation, migration, and maturation, ultimately supporting functional recovery [20]. Emerging evidence indicates that angiogenesis is positively associated with improved motor/sensory recovery in animal models and correlates with increased survival rates in stroke patients [21, 22]. Prior research demonstrates that NBP facilitates functional recovery post‐ischemic stroke by stimulating angiogenesis [9]. Consistent with prior research, our study revealed that NBP treatment not only increased microvessel density by CD31 labeling and gelatin‐ink perfusion but also actively promoted new microvessels' formation in the peri‐infarct cortex, as evidenced by BrdU^+^/CD31^+^ co‐labeling. Moreover, these alterations in microvascular parameters were positively associated with neurological recovery on day 14 post‐stroke. Pericytes and astrocytes are critically required for vascular stabilization, BBB integrity, and vascular remodeling during stroke recovery [23, 24, 25, 26, 27]. Once nascent vessels are formed, pericytes are recruited and retained to the vascular wall, contributing to vessel stability [25]. During vascular remodeling after stroke, astrocytes provide structural support by contacting or wrapping around new vessel sprouts with their end‐feet, ensuring the stability of the vessels [27]. Our results demonstrate that NBP treatment enhances the coverage of microvessels by astrocytic endfeet and pericytes. Collectively, these findings indicate that NBP administration robustly promotes cerebral angiogenesis and improves functional outcomes in subacute‐phase stroke recovery.

The PI3K/Akt signaling pathway plays a central role in regulating angiogenesis through its downstream effectors [28]. Upon activation, phosphorylated PI3K triggers the activation of Akt and subsequently modulates the activity of downstream targets critical for endothelial cell function and angiogenesis, such as mTOR [29], HIF‐1α [30, 31], eNOS [32], and GSK‐3β [15]. GSK‐3β phosphorylates β‐catenin, leading to its proteasomal degradation, while GSK‐3β inhibition stabilizes β‐catenin and promotes its nuclear translocation to activate transcription of pro‐angiogenic genes [33, 34, 35]. Emerging evidence indicates that drugs targeting the Akt/GSK‐3β/β‐catenin signaling pathway enhance post‐stroke angiogenesis and facilitate functional recovery [13, 15]. As the most representative pro‐angiogenic factor, VEGF promotes new blood vessel formation by stimulating vascular sprouting, endothelial cell proliferation, migration, and lumen formation, and blood vessel maturation [36, 37]. Previous studies have confirmed that VEGF is also a key downstream target gene of β‐catenin [38, 39, 40]. In a mouse hindlimb ischemia model, β‐catenin markedly enhanced blood perfusion recovery, augmented capillary density, upregulated VEGF expression, and promoted the proliferation of both endothelial cells and myocytes [38]. Current evidence indicates that NBP promotes post‐stroke angiogenesis through multiple pathways, including Shh and HIF‐1α/VEGF signaling [9, 10, 41], however, the involvement of the Akt/GSK‐3β/β‐catenin pathway remains to be elucidated. Our results demonstrated that NBP activated the Akt/GSK‐3β/β‐catenin pathway and upregulated VEGF expression, both of which were reversed by LY294002. Furthermore, NBP‐induced angiogenesis and neurological function enhancement were also abolished by LY294002. This study presents the first demonstration that NBP promotes post‐stroke angiogenesis and functional recovery through Akt/GSK‐3β‐mediated VEGF upregulation.

Blood flow reestablishment in the peri‐infarct zone correlates with behavioral improvement, likely mediated through two mechanisms: first, resolving metabolic stress to restore neuronal function in previously hypoperfused tissue, and second, ensuring adequate blood flow essential for neural repair, including behavior‐related synaptic remodeling [24]. Speckle imaging enables real‐time observation of cortical blood flow changes during ischemic events [42, 43]. The TPMI technique allows for tracking dynamic microvascular structural alterations with high clarity post‐stroke, permitting detailed analysis of vessel diameter and capillary density variations [44]. Using these techniques, our investigation revealed that NBP therapy improved cortical perfusion, enhanced vasodilation, and promoted neurological recovery on day 14 post‐stroke, all of which were inhibited by LY294002. An important observation was the delayed nature of this perfusion improvement, which became statistically significant only on day 14. While a positive trend was noted on day 7, it did not reach significance. This temporal profile suggests that NBP's primary mechanism is the promotion of angiogenic remodeling. This hypothesis is strongly supported by our histological findings, which demonstrated a significant increase in microvessel density and coverage of astrocytes and pericytes in the peri‐infarct region on day 14. Angiogenesis is a time‐dependent process [1, 45, 46, 47]; the significant CBF improvement on day 14 likely reflects the functional integration of these newly formed vessels, which were perhaps still too immature to impact bulk perfusion on the earlier day 7 time point. Clinically, this positions NBP as a promising adjunctive therapy that targets the subacute phase of stroke. Unlike acute reperfusion strategies, which have a narrow time window, NBP's mechanism of promoting vascular remodeling and maturation suggests its therapeutic benefit could extend into the days and weeks following the initial event, offering a new avenue to improve subacute functional prognosis for stroke patients.

The present study has certain limitations. As angiogenesis is regulated by multiple pathways, NBP likely acts through additional pathways beyond Akt/GSK‐3β. Potential crosstalk with other known pathways such as Shh warrants further investigation. Given the potential off‐target effects of pharmacological inhibitors like LY294002, future studies using genetic models, such as endothelial‐specific Akt knockout mice, are warranted to provide more definitive evidence for causality.

## 5 Conclusion

In summary, this study demonstrates that NBP fosters functional neurological recovery after stroke by stimulating angiogenesis, ensuring vessel maturation, and improving vascular function. We identify the Akt/GSK‐3β pathway as a crucial mechanistic driver for this process. These findings position NBP as a promising candidate for subacute stroke intervention, targeting the critical window of neurovascular remodeling.

## Author Contributions

L.Z.: writing – original draft, conceptualization, validation, visualization; S.W.: validation; J.Z. and R.C.: data curation; J.M. and L.W.: writing – review and editing; P.Z.: formal analysis; W.S. and R.X.: validation; X.Z. and C.Z.: funding acquisition, supervision.

## Funding

This work was supported by the National Natural Science Foundation of China, 82271366. Hebei Province Medical Science Research Key Project, 20230062. Natural Science Foundation of Hebei Province, H2021206366.

## Ethics Statement

All procedures were approved by the Animal Care and Use Committee of the Second Hospital of Hebei Medical University (Permit No. HMUSHC‐130318) and the National Institutes of Health Guide for the Care and Use of Laboratory Animals (NIH Publication No. 80‐23, revised in 1996).

## Conflicts of Interest

The authors declare no conflicts of interest.

## Supporting information


**Figure S1:** LY294002 did not cause significant vascular alterations in the healthy brain of sham animals. (A) Representative CD31^+^ microvascular images on day 14. (B) Quantitative analysis of microvessel density, total vessel length, and branching index (*n* = 6). (C) Gelatin‐ink angiograms of the ischemic penumbra area on day 14. (D) Quantitative analysis of microvessel density (*n* = 5). (E) Representative immunofluorescence images of CD31 (red) and GFAP (green) on day 14 post‐stroke. (F) Quantitative analysis of astrocytic coverage around microvessels (*n* = 4). (G) Representative immunofluorescence images of CD31 (green) and PDGFR2 (red) on day 14 post‐stroke. (H) Quantitative analysis of pericytes coverage around microvessels (*n* = 4).

**Table S1:** Antibodies used for Immunofluorescence staining.
**Table S2:** Antibodies used for western blotting.

## Data Availability

The data that support the findings of this study are available from the corresponding author upon reasonable request.
