# Supplementary figures and images for: Dl‐3‐n‐Butylphthalide Promotes Cortical Angiogenesis via Akt/GSK‐3β Signaling in Ischemic Stroke Mice
Source: CNS Neurosci Ther. 2025 Dec 10;31(12):e70698. doi: 10.1002/cns.70698 (PMC12695697; doi:10.1002/cns.70698)

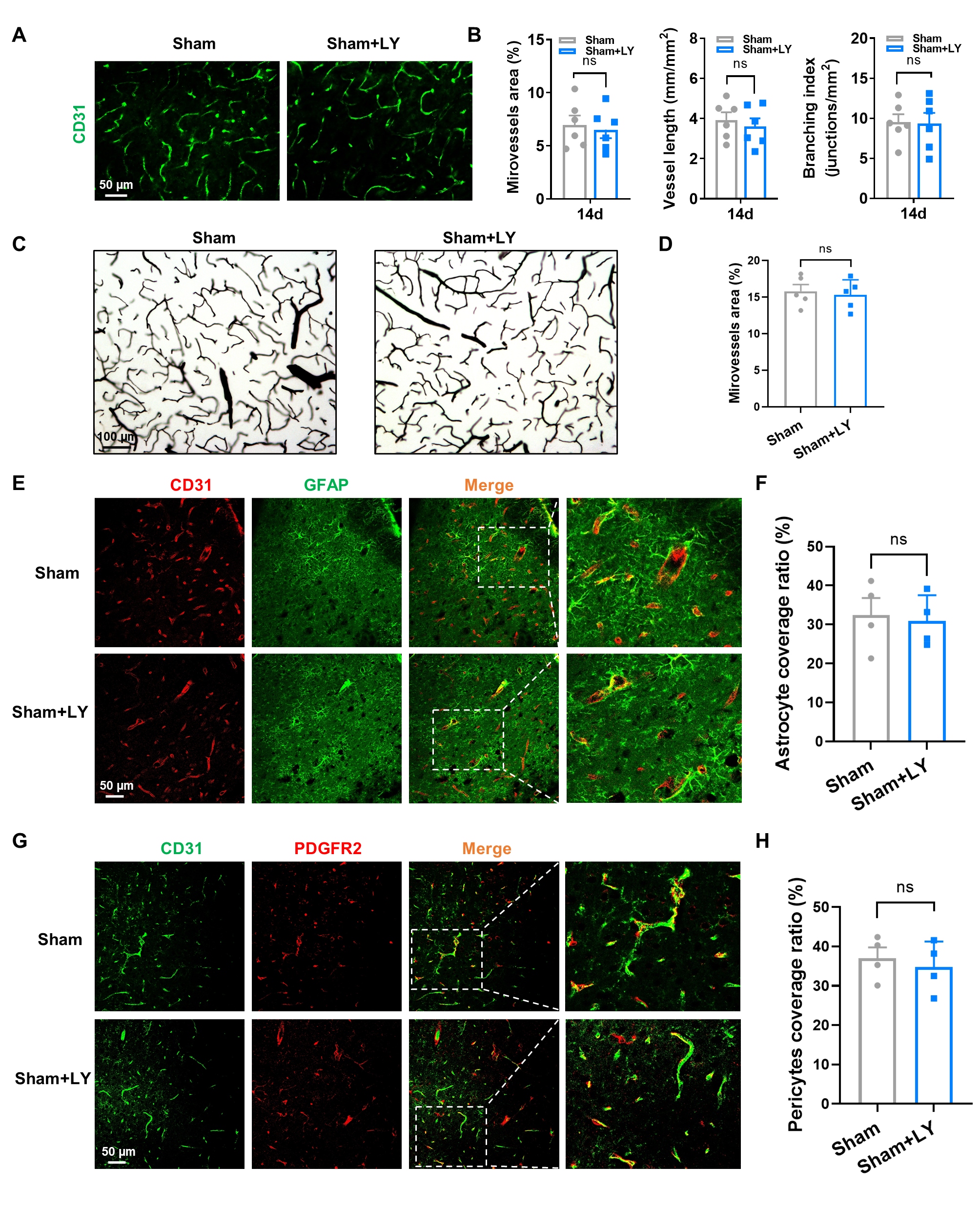

Supplement: Supplementary file 1 — Figure S1: LY294002 did not cause significant vascular alterations in the healthy brain of sham animals. (A) Representative CD31+ microvascular images on day 14. (B) Quantitative analysis of microvessel density, total vessel length, and branching index (n = 6). (C) Gelatin‐ink angiograms of the ischemic penumbra area on day 14. (D) Quantitative analysis of microvessel density (n = 5). (E) Representative immunofluorescence images of CD31 (red) and GFAP (green) on day 14 post‐stroke. (F) Quantitative analysis of astrocytic coverage around microvessels (n = 4). (G) Representative immunofluorescence images of CD31 (green) and PDGFR2 (red) on day 14 post‐stroke. (H) Quantitative analysis of pericytes coverage around microvessels (n = 4). [file CNS-31-e70698-s001.tif]
