# Supplementary material for: Dl‐3‐n‐Butylphthalide Promotes Cortical Angiogenesis via Akt/GSK‐3β Signaling in Ischemic Stroke Mice
Source: CNS Neurosci Ther. 2025 Dec 10;31(12):e70698. doi: 10.1002/cns.70698 (PMC12695697; doi:10.1002/cns.70698)
Supplement: Supplementary file 2 — Table S1: Antibodies used for Immunofluorescence staining. Table S2: Antibodies used for western blotting. [file CNS-31-e70698-s002.docx]

**Table S1. Antibodies used for** **Immunofluorescence staining.**

| **Primary Antibodies** | **Host Species** | **Supplier** | **Catalog Number** | **Dilution** |
| --- | --- | --- | --- | --- |
| BrdU | sheep | Abcam | ab1893 | 1:200 |
| CD31 | rat | BD Biosciences | #550274 | 1:50 |
| GFAP | mouse | EMD Millipore | MAB360 | 1:450 |
| PDGF Receptor β | rabbit | Cell Signaling Technology | #3169 | 1:100 |

**Table S2. Antibodies used for western blotting.**

| **Primary Antibodies** | **Host Species** | **Supplier** | **Catalog Number** | **Dilution** |
| --- | --- | --- | --- | --- |
| p-Akt (Ser473) | Rabbit | Cell Signaling Technology | #9271 | 1:500 |
| Akt | Rabbit | Cell Signaling Technology | #9272 | 1:500 |
| p-GSK-3β (Ser9) | Rabbit | Cell Signaling Technology | #9323 | 1:500 |
| GSK-3β | Rabbit | Santa Cruz Biotechnology | sc-9166 | 1:200 |
| β-catenin | Rabbit | Cell Signaling Technology | #9562 | 1:500 |
| VEGF | Rabbit | Santa Cruz Biotechnology | sc-152 | 1:200 |
| β-actin | mouse | Santa Cruz Biotechnology | sc-47778 | 1:800 |
